# A robust anonymous biometric-based authenticated key agreement scheme for multi-server environments

**DOI:** 10.1371/journal.pone.0187403

**Published:** 2017-11-09

**Authors:** Hua Guo, Pei Wang, Xiyong Zhang, Yuanfei Huang, Fangchao Ma

**Affiliations:** 1 Beijing Key Laboratory of Network Technology, Beihang University, Beijing 100191, China; 2 National computer network and information security laboratory, National Computer network Emergency Response technical Team/Coordination Center, Beijing 100029, China; 3 State Key Laboratory of Space-Ground Integrated Information Technology, Beijing 100020, China; 4 Beijing information technology institute, Beijing 100094, China; King Saud University, SAUDI ARABIA

## Abstract

In order to improve the security in remote authentication systems, numerous biometric-based authentication schemes using smart cards have been proposed. Recently, Moon *et al.* presented an authentication scheme to remedy the flaws of Lu *et al.*’s scheme, and claimed that their improved protocol supports the required security properties. Unfortunately, we found that Moon *et al.*’s scheme still has weaknesses. In this paper, we show that Moon *et al.*’s scheme is vulnerable to insider attack, server spoofing attack, user impersonation attack and guessing attack. Furthermore, we propose a robust anonymous multi-server authentication scheme using public key encryption to remove the aforementioned problems. From the subsequent formal and informal security analysis, we demonstrate that our proposed scheme provides strong mutual authentication and satisfies the desirable security requirements. The functional and performance analysis shows that the improved scheme has the best secure functionality and is computational efficient.

## 1 Introduction

Nowadays security has becoming an urgent issue for the distributed networks. The remote user authentication scheme allows the transmission of secret data via public channels, thus is an important cryptographic tool for distributed networks. In 1981, Lamport [1] proposed the first password-based authentication scheme. After that, considerable amount of work on password-based authentication schemes have been put forward for different applications [2, 3]. However, passwords are vulnerable to be broken in a short time by using dictionary guessing attack. To solve this problem, smart cards with password-based authentication schemes [4–12] are introduced to enhance the security of user authentication. Unfortunately, there are still some problems when the smart card is stolen and the stored data is leaked [13–15].

The biometric keys, such as fingerprint and iris, are considered to be a unique identifier of a user, thus have many advantages. For example, the biometric keys cannot be forgotten or lost, are difficult to copy or share, and are not easy to forge or guess. Additionally, one can carry biometric keys at anytime and from anywhere. With the security requirements of the distributed networks and the good security performance and advantages of the biological characteristic, biometrics authentication protocols come to be more crucial and widely deployed [16–36]. In 2002, Lee *et al.* [16] designed the first biometrics-based remote user authentication scheme. In 2004, Lin-Lai [17] demonstrated that Lee *et al.*’s scheme cannot resist impersonation attack and designed a protocol without verification table to fix the flaws of Lee *et al.*’s scheme. In 2007, Khang-Zhang [18] pointed out that Lin-Lai’s scheme is insecure against server spoofing attack and illustrated an improved scheme. Rhee [19] demonstrated that Khang-Zhang’s scheme is vulnerable to impersonation attack and offline password guessing attack. Later, Li-Wang [20] designed an efficient three-factor remote user authentication scheme which only uses symmetric cryptographic primitive and the hash operation. However, in 2011, Das [21] exhibited that Li-Wang’s scheme is insecure against man-in-the-middle attack and does not provide proper certification. Furthermore, he designed a new certification scheme based on biometric characteristics. In 2014, Li *et al.* [25] pointed out that Das *et al.*’s scheme is vulnerable to forgery attack and stolen smart card attack, and put forward a three-factor remote user authentication scheme. After that, Chaturvedi *et al.* [26] demonstrated that Li *et al.*’s scheme doesn’t resist known session specific temporary information attack and doesn’t protect user’s privacy. They also proposed a novel authentication and key agreement protocol to overcome the weaknesses of Li *et al.*’s scheme.

In 2014, Chuang-Chen [27] proposed an efficient lightweight three-factor authentication protocol for multi-server environment which requires only the hash operation. After that, Mishra *et al.* [28] showed that Chuang-Chen’s scheme is insecure against the denial-of-service attack, smart card stolen attack, server spoofing attack and impersonation attack. In addition, they proposed a new biometric-based multi-server authentication protocol so as to overcome the weaknesses of Chuang-Chen’s scheme. In 2015, Lu *et al.* [29] illustrated that Mishra *et al.*’s scheme is insecure against server spoofing attack and impersonation attack, and can not provide forward secrecy. They introduced two independent three-factor authentication schemes [29, 31] for multi-server architecture, and claimed that the improved scheme has strong security. Unfortunately, Moon *et al.* [30] showed that Lu *et al.*’s scheme [29] is vulnerable to outsider attack and user impersonation attack, and put forward an enhanced protocol which fixes the flaws of Lu *et al.*’s scheme.

Unfortunately, we found that Moon *et al.*’s biometric-based remote user authentication scheme still has some flaws. In this paper, we firstly showed that Moon *et al.*’s scheme is vulnerable to insider attack, server spoofing attack, user impersonation attack and guessing attack. Moreover, we exhibited that their scheme is not anonymous for the user. Then we proposed an improved authentication scheme for multi-server environment to fix their design flaws. After that, we show that our scheme is robust against all known attacks through the formal and informal security analysis. Finally we demonstrate that the improved scheme has the best secure functionality and is computational efficient.

The rest of the paper is organized as follows. In section 2, we introduce some preliminary knowledge. Section 3 briefly reviews Moon *et al.*’s biometric-based remote user authentication scheme. Section 4 shows the design flaws in Moon *et al.*’s scheme. In order to eliminate the shortcomings discussed in section 4, we propose an enhancement authentication protocol in section 5. Section 6 analyzes the security of the proposed scheme, and Section 7 compares the performance of the enhanced scheme with other related schemes. Finally, we conclude in section 8.

## 2 Preliminaries

This section elaborates the definitions of one-way hash function and BioHashing, and the security model.

### 2.1 Definition

**One-way hash function.** A one-way hash function *h*: {0, 1}* → {0, 1}^*n*^ takes an arbitrary-length input *x* ∈ {0, 1}*, and produces a fixed-length output *h*(*x*) ∈ {0, 1}^*n*^, called the message digest. The hash function has the following attributes:

- Computationally, it is easy to compute *y* = *h*(*x*) if *x* and *h*(⋅) are specified.
- It is almost impossible through polynomial time *t* to know two inputs *x*_1_ and *x*_2_, such that *h*(*x*_1_) = *h*(*x*_2_).

**BioHashing.** BioHashing technique [37] is designed to reduce the probability of denial of access while keeping the false acceptation performance. Inputing the biometric feature set and a seed which represents the “Hash key”, BioHashing generates a vector of bits. More precisely, with the help of a uniform distributed pseudo-random numbers generated by giving a secret seed, the biometric vector data *x* ∈ *R*^*n*^ is reduced down to a bit vector *b* ∈ {0, 1}^*l*^ with *l* the length of the bit string (*l* ≤ *n*) through BioHashing.

### 2.2 Security model

In this paper, we adopt the security model proposed by Abdalla et al. [38] to prove the security of our protocol.

- **Participants.** An oracle $\pi_{S_{j}}^{t}$ denotes an instance *t* of a party *S*_*j*_, $\pi_{U_{i}}^{u}$ denotes the instance *u* of *U*_*i*_, and $\pi_{RS}^{v}$ denotes the instance *v* of *RS*.
- **Partnering.** The partner of an instance $\pi_{U_{i}}^{u}$ of *U*_*i*_ is the instance $\pi_{S_{j}}^{t}$ of *S*_*j*_ and conversely. The partial transcript of all exchanged messages between *U*_*i*_ and *S*_*j*_ is unique, and is said as a session ID $sid_{Ui}^{u}$ for the present session in which $\pi_{U_{i}}^{u}$ participates.
- **Freshness.** $\pi_{S_{j}}^{t}$ or $\pi_{U_{i}}^{u}$ is fresh, only if the session key *SK* is not leaked to A.
- **Adversary.** In the ROR model, A models the real attack via the following oracle queries. To breach the security of the authentication protocol, A is able to access the queries given below:
  - *Execute*(*π*^*t*^, *π*^*u*^): The *Execute* query helps A obtain the messages transmitted between two honest participants; this query models an eavesdropping attack.
  - *Send*(*π*^*t*^; *x*): The *Send* query corresponds to an active attack. *π*^*t*^ executes the protocol and responds with an outgoing message after receiving a message *x* from A.
  - *Reveal*(*π*^*t*^): The A executes *Reveal* query to reveal of session keys. If the session has been accepted, *π*^*t*^ returns the session key *SK* as its response that is computed between *π*^*t*^ and its partner, otherwise returns a null value.
  - *CorruptSC*(*π*^*t*^): It is about modeling smart card loss attack and outputs the information stored in *SC*_*i*_.
  - *Test*(*π*^*t*^): At some point, the adversary A can make a Test query to an oracle Π^*t*^. Π^*t*^ flips an unbiased coin *b* and responds with the real agreed session key *SK* if *SK* is established and fresh, if *b* = 1; otherwise it returns a random sample generated according to the distribution of the session key. Otherwise, it returns ⊥.

**Semantic security of the session key.** In an experiment, the adversary A is challenged to differentiate between an instance’s real session key *SK* and a random key. A can continue querying *Test* queries to either the server instance or the user instance. The outcome of *Test* query must be consistent with the random bit *b*. Eventually, A terminates the game simulation and outputs a bit *b*′ for *b*. we say A wins if the adversary guesses the correct *b*.

Let *E* denotes the event that A wins the game. Then, the advantage of A breaches the semantic security of our proposed authenticated key-agreement (AKE) protocol, say P, is computed as $Adv_{P}^{ake}\left(A\right)=\left|2pr\left[E_{0}\right]-1\right|$. We say that the protocol P is a secure multi-server authentication and key agreement protocol in the ROR sense if $Adv_{P}^{ake}$ is negligible.

**Random oracle.** To prove the security of the proposed protocol, the one-way hash function *h*(⋅) is treated as a random oracle(say Hash oracle), and is provided to the adversary A and every participant. The Hash oracle is simulated by a two-tuple (*u*, *v*) table of binary strings. When a hash query *h*(*u*) is made, the *Hash* oracle returns *v* if *u* is found in the table; otherwise, it returns a uniformly random string *v* and stores the pair (*u*, *v*) in the table.

## 3 Review of Moon *et al.*’s scheme

In this section, we briefly review Moon *et al.*’s scheme, which consists of four phases: registration phase, login phase, authentication phase and password change phase. Table 1 summarizes the notations used in this paper.

**Table 1:** Notations.

Notations	Description
*U*_*i*_	An *i*_*th*_ user
*AS*	Application server
*RS*	Registration server
*ID*_*i*_	Identity of *U*_*i*_
*PW*_*i*_	Password of *U*_*i*_
*SC*	smart card
*SID*_*j*_	Identity of *AS*
*PSK*	Secret keys chosen by *RS* for *AS*
*E*{}, *D*{}	Encryption and decryption operations
$P_{ub_{s}}$, $P_{ri_{s}}$	Public and private keys of *AS*
*n*_1_, *n*_2_	Random numbers chosen by *U*_*i*_ and *AS*
*h*(⋅)	A secure one-way hash function
*H*(⋅)	A bio-hash function
⊕	An exclusive-OR operation
||	The concatenation operation

Table 1 summarizes the notations used in this paper.

### 3.1 Registration phase

**Fig 1:**
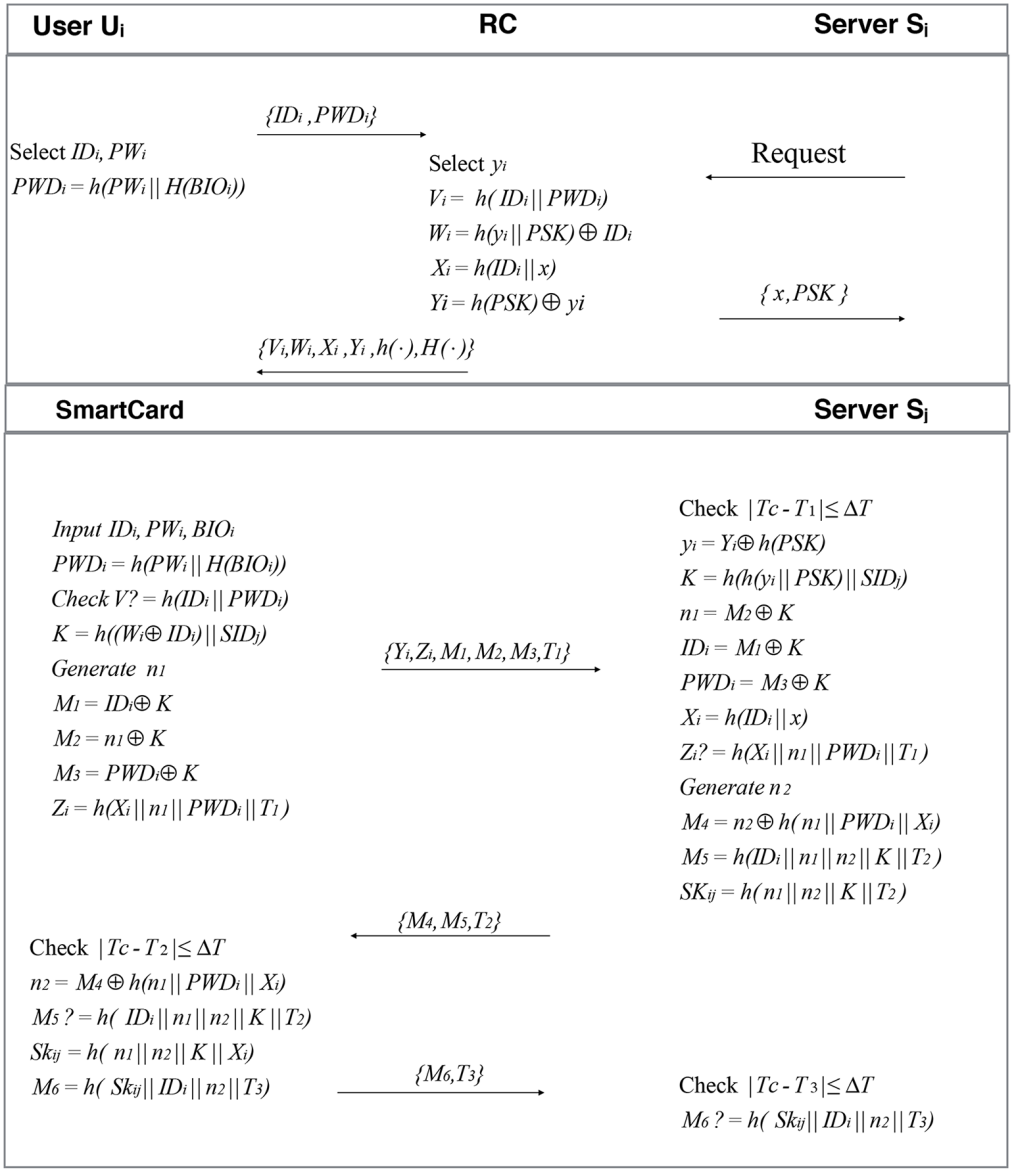
Registration and authentication phases of Moon *et al.*’s scheme. Registration and authentication phases of Moon *et al.*’s scheme.

The registration and authentication phases are shown in Fig 1. In order to get the access to different services provided by the servers, a user must register himself through the registration server. *U*_*i*_ firstly selects an identity *ID*_*i*_ and password *PW*_*i*_ and inputs biometrics *BIO*_*i*_.

1. Using the password and the biometrics, the smart card computes *PWD*_*i*_ = *h*(*PW*_*i*_||*H*(*BIO*_*i*_)) and sends < *ID*_*i*_, *PWD*_*i*_ > to the registration server through a secure channel.
2. Upon receiving the message < *ID*_*i*_, *PWD*_*i*_ >, the registration server computes *V*_*i*_ = *h*(*ID*_*i*_||*PWD*_*i*_), *W*_*i*_ = *h*(*y*_*i*_||*PSK*) ⊕ *ID*_*i*_, *X*_*i*_ = *h*(*ID*_*i*_||*x*), *Y*_*i*_ = *y*_*i*_⊕ *h*(*PSK*). Then *RS* stores < *V*_*i*_, *W*_*i*_, *X*_*i*_, *Y*_*i*_, *h*(⋅), *H*(⋅) > onto a smart card and sends the smart card to *U*_*i*_.

### 3.2 Login phase

During the login phase, the user *U*_*i*_ inserts his smart card into the smart card reader, inputs his identity *ID*_*i*_ and password *PW*_*i*_, and imprints biometric information *BIO*_*i*_. Upon receiving an input, the smart card uses the following steps to perform a login session:

1. The smart card computes *PWD*_*i*_ = *h*(*PW*_*i*_||*H*(*BIO*_*i*_)) and verifies *V*_*i*_? = *h*(*ID*_*i*_|| *PWD*_*i*_). If succeeds, it executes the next step. Otherwise the session aborts.
2. The smart card generates a random number *n*_1_ and computes *K* = *h*((*W*_*i*_ ⊕ *ID*_*i*_)|| *SID*_*j*_), *M*_1_ = *ID*_*i*_ ⊕ *K*, *M*_2_ = *n*_1_ ⊕ *K*, *M*_3_ = *PWD*_*i*_ ⊕ *K*, *Z*_*i*_ = *h*(*X*_*i*_||*n*_1_|| *PWD*_*i*_||*T*_*i*_).
3. The smart card transmits the login request message < *Y*_*i*_, *Z*_*i*_, *M*_1_, *M*_2_, *M*_3_, *T*_1_ > to the server *S*_*j*_ through a public channel, where *T*_1_ is the current timestamp.

### 3.3 Authentication phase

After receiving the authentication request < *Y*_*i*_, *Z*_*i*_, *M*_1_, *M*_2_, *M*_3_, *T*_1_ > from the user *U*_*i*_, the server *S*_*j*_ executes the following steps to authenticate each other.

1. The server *S*_*j*_ firstly checks whether |*T*_*c*_ − *T*_1_| < Δ*T*, then uses its pre-shared key *PSK* and achieves *y*_*i*_ = *Y*_*i*_ ⊕ *h*(*PSK*). The server also retrieves *K* = *h*(*h*(*y*_*i*_||*PSK*)||*SID*_*j*_), *n*_1_ = *M*_2_ ⊕ *K*, *ID*_*i*_ = *M*_1_ ⊕ *K*, *PWD*_*i*_ = *M*_3_ ⊕ *K*, *X*_*i*_ = *h*(*ID*_*i*_||*x*) and verifies *Z*_*i*_? = *h*(*X*_*i*_||*n*_1_||*PWD*_*i*_||*T*_1_). If they are not equal, *S*_*j*_ rejects the login request and terminates the session. Otherwise, the server generates a random number *n*_2_ and computes *M*_4_ = *n*_2_ ⊕ *h*(*n*_1_||*PWD*_*i*_||*X*_*i*_), *M*_5_ = *h*(*ID*_*i*_||*n*_1_||*n*_2_||*K*||*T*_2_), *SK*_*ij*_ = *h*(*n*_1_||*n*_2_||*K*||*T*_2_) and then responds with the message < *M*_4_, *M*_5_, *T*_2_ > to the smart card (user *U*_*i*_) over a public channel.
2. Upon receiving the message < *M*_4_, *M*_5_, *T*_2_ > and checking the freshness of *T*_2_, the smart card retrieves the value *n*_2_ = *M*_4_ ⊕ *h*(*n*_1_||*PWD*_*i*_||*X*_*i*_). Then it verifies *M*_5_? = *h*(*ID*_*i*_||*n*_1_||*n*_2_||*K*||*T*_2_). If the verification holds, it computes the session key *SK*_*ij*_ = *h*(*n*_1_||*n*_2_||*K*||*X*_*i*_), which would be shared between *U*_*i*_ and *S*_*j*_. Finally, the smart card computes *M*_6_ = *h*(*SK*_*ij*_||*ID*_*i*_||*n*_2_||*T*_3_) and sends the message < *M*_6_, *T*_3_ > to *S*_*j*_ via a public channel.
3. Upon receiving the message < *M*_6_, *T*_3_ >, *S*_*j*_ checks the freshness of *T*_3_ and verifies *h*(*SK*_*ij*_||*ID*_*i*_||*n*_2_||*T*_3_)? = *M*_6_. If the equation holds, the server ensures the identity of *U*_*i*_. Otherwise, the server aborts the session.

### 3.4 Password updating

In this phase, *U*_*i*_ can change his password any time when he wants. In order to change password, the user performs the following steps:

1. *U*_*i*_ inserts his smart card into the smart card reader and then inputs *ID*_*i*_ and *PW*_*i*_ and biometrics *BIO*_*i*_.
2. The smart card *SC*_*i*_ computes *PWD*_*i*_ = *h*(*PW*_*i*_||*H*(*BIO*_*i*_)), then checks if *Vi*′ = *h*(*ID*_*i*_||*PWD*_*i*_) is the same as the stored *V*_*i*_. If they are the same, *SC*_*i*_ accepts *U*_*i*_ to enter a new password $PW_{i_{new}}$.
3. *SC*_*i*_ computes $PWD_{i_{new}}=h(PW_{i_{new}}||H\left(BIO_{i}\right))$ and $V_{i_{new}}=h(ID_{i}||PWD_{i_{new}})$, and replaces *V*_*i*_ with $V_{i_{new}}$.

## 4 Security analysis of Moon *et al.*’s scheme

Although Moon *et al.* claimed that their scheme satisfies the required security requirements, we found that their scheme still has some weakness, i.e., fail to resist the insider attack, server spoofing attack, guessing attack and impersonation attack. Moreover, their scheme is not anonymous for users.

### 4.1 Lack of user anonymity

User anonymity means that the adversary cannot obtain or track the identity of the user according to the message transmitted via the public channel, which is an important property to protect the privacy of users. In Moon *et al.*’s scheme, during authentication phase, *U*_*i*_ sends < *Y*_*i*_, *Z*_*i*_, *M*_1_, *M*_2_, *M*_3_, *T*_1_ > as authentication request message to *S*_*j*_. Note that all the information transmitted in public channel can be intercepted by the adversary. The parameter *M*_1_ = *K* ⊕ *ID*_*i*_ where *K* = *h*((*W*_*i*_ ⊕ *ID*_*i*_)||*SID*_*j*_)) in the message < *Y*_*i*_, *Z*_*i*_, *M*_1_, *M*_2_, *M*_3_, *T*_1_ >, is unique and static for each user during all logins to the same server. Thus anyone has ability to track the activities of a legal user, if he captures the value of *M*_1_.

### 4.2 Insider attack

Insider attack means that an insider can get the sensitive credentials from the information stored in *RS*. In Moon *et al.*’s scheme, during user registration phase, *U*_*i*_ submits his identity *ID*_*i*_ and *PWD*_*i*_ to *RS*. In order to prevent duplicate user registration, *RS* has to store the user’s *ID*. If an adversary obtains the list of *ID*, it would cause great devastation. The adversary can impersonate himself as *U*_*i*_ as described in the following user impersonation attack.

### 4.3 Server spoofing attack

In Moon *et al.*’s protocol, *RS* shares the same secret information (*x*, *PSK*) with all the application severs. The compromised sever can impersonate as another legitimate server to deceive any legal user. Now we show the reason why Moon *et al.*’s scheme cannot withstand this kind of server spoofing attack.

1. When *U*_*i*_ submits his login request message < *Y*_*i*_, *Z*_*i*_, *M*_1_, *M*_2_, *M*_3_, *T*_1_ > to *S*_*j*_, the legal but malicious server *S*_*k*_ can intercept this message and compute *y*_*i*_ = *Y*_*i*_ ⊕ *h*(*PSK*), *K* = *h*(*h*(*y*_*i*_||*PSK*)||*SID*_*j*_), *n*_1_ = *M*_2_ ⊕ *K*, *ID*_*i*_ = *M*_1_ ⊕ *K*, *PWD*_*i*_ = *M*_3_ ⊕ *K*, *X*_*i*_ = *h*(*ID*_*i*_||*x*) and to check *Z*? = *h*(*X*_*i*_||*n*_1_||*PWD*_*i*_||*T*_1_).
2. *S*_*k*_ generates a random number *n*_2_ and computes *M*_4_ = *n*_2_ ⊕ *h*(*n*_1_||*PWD*_*i*_||*X*_*i*_), *M*_5_ = *h*(*ID*_*i*_||*n*_1_||*n*_2_||*K*||*T*_2_), *SK*_*ij*_ = *h*(*n*_1_||*n*_2_||*K*||*T*_2_), then sends < *M*_4_, *M*_5_, *T*_2_ > to *U*_*i*_.
3. *U*_*i*_ computes *n*_2_ = *M*_4_ ⊕ *h*(*n*_1_||*PWD*_*i*_||*X*_*i*_), *M*_5_ = *h*(*ID*_*i*_||*n*_1_||*n*_2_||*K*||*T*_2_) and compares it with *M*_5_. It is obvious that the values are the same, thus *U*_*i*_ responds with the message *M*_6_ = *h*(*SK*_*ij*_||*ID*_*i*_||*n*_2_||*T*_3_).
4. *U*_*i*_ computes the session key *SK*_*ij*_ = *h*(*n*_1_||*n*_2_||*K*||*T*_2_) and believes that he is communicating with *S*_*j*_.

Therefore, a legal but malicious server *S*_*k*_ can masquerade as another server *S*_*j*_ to fool any legal user and Moon *et al.*’s scheme is vulnerable to server spoofing attack.

### 4.4 Guessing attack

Moon *et al.*’s scheme is vulnerable to identity guessing attack, which is a critical concern in their scheme. If the adversary can extract the secret value *W*_*i*_ from the legal user’s smart card by some means and get the value of *M*_1_ from public channel, the adversary can easily find out $ID_{i}^{*}$ by performing the guessing attack, in which each guess *ID*_*i*_ can be verified as the following steps.

1. The adversary chooses $ID_{i}^{*}$ and computes $K=h(\left(W_{i}⊕ID_{i}^{*}\right)||SID_{j})$.
2. The adversary verifies the correctness of $ID_{i}^{*}$ by checking $M_{1}?=ID_{i}^{*}⊕K$.
3. The adversary repeats the above steps until a correct $ID_{i}^{*}$ is found.

### 4.5 User impersonation attack

In a remote user communication scheme, anyone should be considered as a legal user if a user has valid authentication credentials or could be capable of constructing an effective authentication request message. In Moon *et al.*’s protocol, an adversary can impersonate a valid user as described below.

1. As enlightened in insider attack and guessing attack mentioned above, an adversary obtains *U*_*i*_’s personal identifiable information *ID*_*i*_. He also extracts the secret values *W*_*i*_ and *X*_*i*_ from the legal user’s smart card by some means.
2. The adversary intercepts a valid login request message < *Y*_*i*_, *Z*_*i*_, *M*_1_, *M*_2_, *M*_3_, *T*_1_ > which is sent from *ID*_*i*_ via the public channel, then the adversary computes *K* = *ID*_*i*_ ⊕ *M*_1_, *PWD*_*i*_ = *K* ⊕ *M*_3_, chooses random number *n*_1_, and calculates *M*_1*m*_ = *ID*_*i*_ ⊕ *K*, *M*_2*m*_ = *n*_1_ ⊕ *K*, *M*_3*m*_ = *PWD*_*i*_ ⊕ *K*, *Z*_*im*_ = *h*(*X*_*i*_||*n*_1_||*PWD*_*i*_|| $T_{1}^{\prime })$. Now, the malicious adversary sends the forged login request message < *Y*_*i*_, $Z_{im},M_{1m},M_{2m},M_{3m},T_{1}^{\prime }>$ to *S*_*j*_ by masquerading as legal user *U*_*i*_.
3. After the authentication of the login request message, the server *S*_*j*_ generates a random number *n*_2_, computes *M*_4*m*_ = *n*_2_ ⊕ *h*(*n*_1_||*PWD*_*i*_||*X*_*i*_), *M*_5*m*_ = *h*(*ID*_*i*_||*n*_1_ ||*n*_2_||*K*||*T*_2_) and responds with the message < *M*_4*m*_, *M*_6*m*_, *T*_2_ > to the adversary who is masquerading as *U*_*i*_.
4. The masquerading adversary verifies the correctness of *M*_4*m*_ with the values of *n*_1_ and *K*. Then the masquerading user *U*_*i*_ computes *n*_2_ = *M*_4*m*_ ⊕ *h*(*n*_1_||*PWD*_*i*_||*X*_*i*_), *SK*_*ij*_ = *h*(*n*_1_||*n*_2_||*K*||*T*_2_), *M*_6*m*_ = *h*(*SK*_*ij*_||*ID*_*i*_||*n*_2_||*T*_3_), and sends the message < *M*_6*m*_, *T*_3_ > back to the server *S*_*j*_.
5. The server *S*_*j*_ computes *M*_6*m*_ = *h*(*SK*_*ij*_||*ID*_*i*_||*n*_2_||*T*_3_) and verifies it with the received value of *M*_6*m*_. It is obvious that they are equal, so the sever authenticates successfully the legitimacy of the user *U*_*i*_ and the login request message information is accepted.
6. After mutual authentication, the server *S*_*j*_ and the malicious adversary who masquerades as the user *U*_*i*_ agree on the common session key as *SK*_*ij*_ = *h*(*n*_1_|| *n*_2_||*K*||*X*_*i*_).

## 5 Our proposed scheme

**Fig 2:**
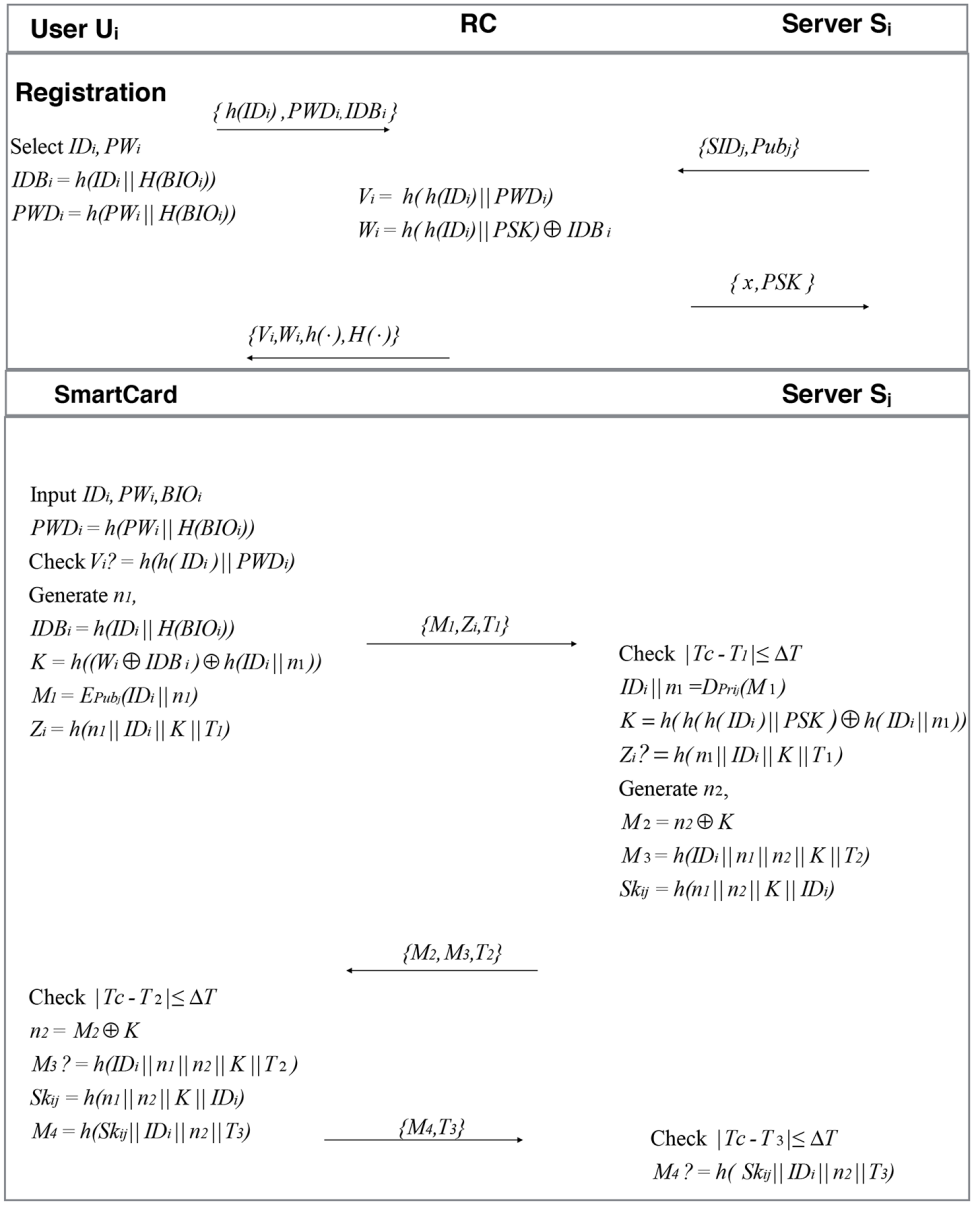
Registration and authentication phases of our scheme. Registration and authentication phases of our scheme.

In this section, we propose an improved remote user authentication scheme to fix the drawbacks in Moon *et al.*’s scheme. Our proposed protocol consists of four phases: registration, login, mutual authentication with key-agreement and password change. Fig 2 describes our proposed scheme.

### 5.1 Registration phase

When the remote user authentication scheme starts, the user *U*_*i*_ and the server *S*_*j*_ need to perform the following steps to register with the registration server(*RS*).

#### 5.1.1 Server registration

To register with the system, a server *S*_*j*_ submits his identity *SID*_*j*_ and his public key *Pub*_*j*_ which can be obtained by all the users. Then *S*_*j*_ sends his identity *SID*_*j*_ and his public key *Pub*_*j*_ to *RS*. Upon reception, *RS* shares the secret key *PSK* with *S*_*j*_ and publishes *S*_*j*_’s public key *Pub*_*j*_.

#### 5.1.2 User registration

1. *U*_*i*_ freely selects his identity *ID*_*i*_ which uniquely identifies the user’s identity, password *PW*_*i*_ and scans his biometrics *BIO*_*i*_. Then *U*_*i*_ computes *IDB*_*i*_ = *h*(*ID*_*i*_ ||*H*(*BIO*_*i*_)), *PWD*_*i*_ = *h*(*PW*_*i*_||*H*(*BIO*_*i*_)) and sends < *h*(*ID*_*i*_), *IDB*_*i*_, *PWD*_*i*_ > to *RS* on a secure channel.
2. Upon reception, *RS* computes *V*_*i*_ = *h*(*h*(*ID*_*i*_)||*PWD*_*i*_), *W*_*i*_ = *h*(*h*(*ID*_*i*_)||*PSK*) ⊕ *IDB*_*i*_ and stores < *V*_*i*_, *W*_*i*_, *h*(⋅), *H*(⋅) > in the smart card *SC*.
3. *RS* sends *SC* to *U*_*i*_ over a secure channel.

### 5.2 Login phase

1. *U*_*i*_ sends the login request by inserting smart card (*SC*), and inputting *ID*_*i*_, *PW*_*i*_ and *BIO*_*i*_.
2. *SC* computes *PWD*_*i*_ = *h*(*PW*_*i*_||*H*(*BIO*_*i*_)) and then checks whether the condition *V*_*i*_? = *h*(*h*(*ID*_*i*_)||*PWD*_*i*_). If the result is negative, the login session can be aborted. Otherwise, *SC* generates a random number *n*_1_ and computes *K* = *h*((*W*_*i*_ ⊕ *IDB*_*i*_) ⊕ *h*(*ID*_*i*_||*n*_1_)), $M_{1}=E_{Pub_{j}}(ID_{i}\left|\right|n_{1})$, *Z*_*i*_ = *h*(*n*_1_||*ID*_*i*_||*K*||*T*_1_) and sends < *M*_1_, *Z*_*i*_, *T*_1_ > to the server *S*_*j*_ as the login request message.

### 5.3 Authentication phase

1. On getting login message, *S*_*j*_ checks freshness of *T*_1_. *S*_*j*_ computes (*ID*_*i*_||*n*_1_) = $E_{Pri_{j}}\left(M_{1}\right)$, *K* = *h*(*h*(*h*(*ID*_*i*_)||*PSK*) ⊕ *h*(*ID*_*i*_||*n*_1_)) and verifies if *Z*_*i*_? = *h*(*n*_1_|| *ID*_*i*_||*K*||*T*_1_). If they are same, *S*_*j*_ authenticates *U*_*i*_. Otherwise the session is terminated.
2. *S*_*j*_ further generates a random number *n*_2_, and computes *M*_2_ = *n*_2_ ⊕ *K*, *M*_3_ = *h*(*ID*_*i*_||*n*_1_||*n*_2_||*K*||*T*_2_), *SK*_*ij*_ = *h*(*n*_1_||*n*_2_||*K*||*ID*_*i*_). *S*_*j*_ sends < *M*_2_, *M*_3_, *T*_2_ > to *SC*.
3. On checking the freshness of *T*_2_, *SC* computes *n*_2_ = *M*_2_ ⊕ *K* and verifies the condition *M*_3_? = *h*(*ID*_*i*_||*n*_1_||*n*_2_||*K*||*T*_2_). If the condition holds, *U*_*i*_ authenticates *S*_*j*_. Otherwise the process is terminated. Then, *SC* computes *SK*_*ij*_ = *h*(*n*_1_||*n*_2_ ||*K*||*ID*_*i*_) and *M*_4_ = *h*(*SK*_*ij*_||*ID*_*i*_||*n*_2_||*T*_3_), then sends < *M*_4_, *T*_3_ > to *S*_*j*_.
4. *S*_*j*_ checks the freshness of *T*_3_. *S*_*j*_ verifies *M*_4_? = *h*(*SK*_*ij*_||*ID*_*i*_||*n*_2_||*T*_3_) and reconfirms the authenticity of *U*_*i*_. Now, *U*_*i*_ and *S*_*j*_ share with the computed session key *SK*_*ij*_ = *h*(*n*_1_||*n*_2_||*K*||*ID*_*i*_) for further communication.

### 5.4 Password changing phase

This procedure is invoked whenever a user (*U*_*i*_) wants to update his password with a new password $PWD_{i}^{*}$, without through a private channel or communicating with *RS*.

1. *U*_*i*_ inserts smart card *SC* and inputs *ID*_*i*_, *PW*_*i*_ and *BIO*_*i*_.
2. *SC* computes *PWD*_*i*_ = *h*(*PW*_*i*_||*H*(*BIO*_*i*_)) and then verifies the condition *V*_*i*_? = *h*(*ID*_*i*_||*PWD*_*i*_). If the condition doesn’t hold, the request can be dropped.
3. *U*_*i*_ chooses a new password $PW_{i}^{*}$ and then computes $PWD_{i}^{*}=h(PW_{i}^{*}\left|\right|$ $H(BIO_{i}^{*}))$, $V_{i}^{*}=h(h\left(ID_{i}\right)||PWD_{i}^{*})$. Thus the smart card finally contains the parameters $\left\{V_{i}^{*},W_{i},h\left(\cdot \right),H\left(\cdot \right)\right\}$.

## 6 Security analysis of the proposed scheme

In this section, we use Burrows-Abadi-Needham logic (BAN-logic) [39] to verify the completeness of our scheme, then we prove the security of the scheme through formal and informal analysis.

### 6.1 Verifying the proposed scheme with BAN logic

The BAN logic introduced by Burrows *et al.* is a formal method of analyzing the security features of the information exchange protocol. It helps determine whether the exchanged information is credible, whether it can prevent eavesdropping or both. In this paper, we use BAN logic to prove that a user and a server share a session key after successfully running the protocol. We first introduce the BAN logic notations used in this paper in Table 2.

**Table 2:** BAN logic notations.

Notations	Description
*P*| ≡ *X*	*P* believes the statement *X* is true
*P* ⊲ *X*	*P* sees *X*
*P*| ∼ *X*	*P* once said that *X* or has sent a message containing *X*
*P* ⇒ *X*	*P* has control over *X*
#*X*	*X* is fresh
$P\overset{K}{↔}Q$	*P* and *Q* can communicate using the shared key *K*, only *P*, *Q* or a trusted third party know *K*
(*X*)_*k*_	The formula *X* is hashed by *K*
{*X*}_*k*_	The formula *X* is encrypted by *K*
$\overset{K}{\to }S_{j}$	*K* is the public key of *P*, only *P* know the corresponding secret key *K*^−1^

1. BAN logical postulates
  1. Message-meaning rule: $\frac{P|\equiv P\overset{K}{↔}Q,P◁{\left\{X\right\}}_{K}}{P|\equiv Q|∼X}$: If *P* believes that *K* is the shared key of *P* and *Q*, and *P* receives the message *X* encrypted with *K*, then *P* believe that *Q* has sent message *X*.
  2. Jurisdiction rule: $\frac{P|\equiv Q\Rightarrow X,P|\equiv Q|\equiv X}{P|\equiv X}$: If *P* believes that *Q* has the right to control *X* and *P* believes that *Q* also trusts *X*, then *P* trusts *X*.
  3. Nonce-verification rule: $\frac{P|\equiv \#(X),P|\equiv Q|∼X}{P|\equiv Q|\equiv X}$: If *P* believes that *X* is fresh and *P* believes that *Q* has sent *X*, then *P* believes that *Q* believes *X*.
  4. Freshness-conjuncatenation rule: $\frac{P|\equiv \#(X)}{P|\equiv \#(X,Y)}$: If *P* believes that *X* is new, then the information of (*X*, *Y*) is also fresh.
  5. Belief rule: $\frac{P|\equiv X,P|\equiv Y}{P|\equiv (X,Y)}$: If *P* believes *X* and *Y*, then *P* believes (*X*, *Y*).
2. Establishment of security goals
  g1: $S_{j}\left|\equiv U_{i}\right|\equiv U_{i}\overset{SK_{ij}}{↔}S_{j}$
  g2: $S_{j}|\equiv U_{i}\overset{SK_{ij}}{↔}S_{j}$
  g3: $U_{i}\left|\equiv S_{j}\right|\equiv U_{i}\overset{SK_{ij}}{↔}S_{j}$
  g4: $U_{i}|\equiv U_{i}\overset{SK_{ij}}{↔}S_{j}$
3. Initiative premises
  p1. *U*_*i*_| ≡ #*n*_1_. p2. *U*_*i*_| ≡ *S*_*j*_ ⇒ #*n*_2_.
  p3. *S*_*j*_| ≡ #*n*_1_. p4. *S*_*j*_| ≡ #*n*_2_.
  p5. $S_{j}|\equiv U_{i}\overset{K}{↔}S_{j}$. p6. $U_{i}|\equiv U_{i}\overset{K}{↔}S_{j}$.
  p7. *U*_*i*_| ≡ *ID*_*i*_. p8. *S*_*j*_| ≡ *U*_*i*_ ⇒ *ID*_*i*_.
  p9. $S_{j}|\equiv U_{i}\Rightarrow U_{i}\overset{SK_{ij}}{↔}S_{j}$. p10. $U_{i}|\equiv S_{j}\Rightarrow U_{i}\overset{SK_{ij}}{↔}S_{j}$.
4. Scheme analysis
  *a*_0_. $S_{j}◁{\left\{n_{1},ID_{i}\right\}}_{Pub_{j}}$
    Since $\overset{Pri_{j}}{\to }S_{j}$, only *S*_*j*_ can get the value of *ID*_*i*_ and *n*_1_. One can get the value of *K* unless he has the true *Pri*_*j*_ and *PSK* at the same time.
  *a*_1_. *S*_*j*_ ⊲ (*n*_1_, *ID*_*i*_, *T*_1_)_*K*_, *T*_1_
    We employ Message-meaning rule according to *p*_5_ and *a*_1_ to drive:
  *a*_2_. *S*_*j*_| ≡ *U*_*i*_| ∼ (*n*_1_, *ID*_*i*_, *T*_1_)
    According to *a*_2_ and *p*_3_, we apply the Freshness-conjuncatenation rule and Nonce-verification rule to get the following information:
  *a*_3_. *S*_*j*_| ≡ *U*_*i*_| ≡ (*n*_1_, *ID*_*i*_, *T*_1_)
    According to *a*_3_ and *p*_8_, we employ Jurisdiction rule and belief rule to obtain:
  *a*_4_. *S*_*j*_| ≡ *ID*_*i*_
    According to *a*_4_ and $S_{j}◁{\left(U_{i}\overset{SK_{ij}}{↔}S_{j},n_{2},T_{3}\right)}_{ID_{i}},T_{3}$, we employ Message-meaning rule to obtain:
  *a*_5_. $S_{j}\left|\equiv U_{i}\right|∼\left(U_{i}\overset{SK_{ij}}{↔}S_{j},n_{2},T_{3}\right)$
    According to *a*_5_ and *p*_4_, we apply Nonce-verification rule and Freshness- conjuncatenation rule to obtain:
  *a*_6_. $S_{j}\left|\equiv U_{i}\right|\equiv \left(U_{i}\overset{SK_{ij}}{↔}S_{j},n_{2},T_{3}\right)$
    Finally, we employ The belief rule to obtain:
  *g*_1_. $S_{j}\left|\equiv U_{i}\right|\equiv U_{i}\overset{SK_{ij}}{↔}S_{j}$.
    According to *g*_1_ and *p*_9_, we utilize Jurisdiction rule to obtain:
  *g*_2_. $S_{j}|\equiv U_{i}\overset{SK_{ij}}{↔}S_{j}$.
    According to *p*_6_ and *U*_*i*_ ⊲ (*ID*_*i*_, *n*_1_, *n*_2_, *T*_2_)_*K*_, we employ Message-meaning rule to obtain:
  *a*_7_. *U*_*i*_| ≡ *S*_*j*_| ∼ (*ID*_*i*_, *n*_1_, *n*_2_, *T*_2_)
    According to *a*_7_ and *p*_1_ we apply Nonce-verification rule and Freshness- conjuncatenation rule to derive:
  *a*_8_. *U*_*i*_| ≡ *S*_*j*_| ≡ (*ID*_*i*_, *n*_1_, *n*_2_, *T*_2_)
    According to *a*_8_ and *p*_1_, *p*_3_, *p*_4_, *p*_6_ and *SK*_*ij*_ = *h*(*n*_1_||*n*_2_||*K*||*ID*_*i*_), we apply Freshness-conjuncatenation rule and Nonce-verification rule to derive:
  *g*_3_. $U_{i}\left|\equiv S_{j}\right|\equiv U_{i}\overset{SK_{ij}}{↔}S_{j}$.
    According to *g*_3_ and *p*_10_ we utilize Jurisdiction rule to obtain:
  *g*_4_. $U_{i}|\equiv U_{i}\overset{SK_{ij}}{↔}S_{j}$.

### 6.2 Formal analysis

We use provable security to prove the security of our scheme. The security proof is based on the model of RSA-based password authentication.

**Theorem 1.** Let A be an adversary that run in polynomial time *t* against our protocal P in the random oracle, *D* be a uniformly distributed password dictionary and *l* denotes the number of bits in the biometric key *BIO*_*i*_, |*Hash*| and|*D*| denotes the range space of hash function and the size of *D*, respectively. If an attacker A makes *q*_*h*_ *Hash* queries, *q*_*send*_ *Send* queries, then, the advantage of A of breaking the SK-security of P is $Adv_{P}^{ake}\leq \frac{q_{h}^{2}}{\left|Hash\right|}+\frac{q_{send}}{2^{l-1}.\left|D\right|}+2Adv^{RSA}\left(t\right)$, where *Adv*^*RSA*^(*t*) is the advantage that an adversary A solves the problem about the factor decomposed of great number.

**Proof**. The proof is finished by executing a sequence of hybrid games *G*_*i*_. For each game *G*_*i*_, let *E*_*i*_ denote the event that the adversary succeeds in guessing the bit *b* in game *G*_*i*_.

**Game** *G*_0_: This game corresponds to the real attack in the random oracle model. Thus, we can write

$$\begin{array}{c} Adv_{P}^{ake}=\left|2pr\left[E_{0}\right]-1\right| \end{array}$$ (1)

**Game** *G*_1_: By querying *Execute* oracle, this game simulates A’s eavesdropping attack. After that, the adversary queries *Test* oracle, and decides whether the outcome of the *Test* oracle is the real session key *SK* or a random number, where *SK*_*ij*_ is computed from *SK*_*ij*_ = *h*(*n*_1_||*n*_2_||*K*||*ID*_*i*_). Note that *PSK* and *IDB*_*i*_ are secret to *S*_*j*_ and *U*_*i*_. The adversary has no knowledge about *PSK*, *IDB*_*i*_ and *ID*_*i*_, thus eavesdropping of message can not increase the chance of winning for the adversary in *G*_1_. So we have

$$\begin{array}{c} pr\left[E_{0}\right]=pr\left[E_{1}\right] \end{array}$$ (2)

**Game** *G*_2_: The difference between *G*_2_ and *G*_1_ is that we add the simulations of the *Send* and the *Hash* oracles. *G*_2_ models an active attack where A tries to decide a participant into accepting a forged message. A can make several *Hash* queries to find the collisions. Note that the messages {*M*_1_, *Z*_*i*_, *T*_1_} and {*M*_2_, *M*_3_, *T*_2_} are associated with timestamp *T*_1_, *T*_2_, random numbers *n*_1_ and *n*_2_, and *ID*_*i*_ of *U*_*i*_, hence there is no collision when querying the *Send* oracle. According to the birthday paradox, we have

$$\begin{array}{c} |pr\left[E_{2}\right]-pr\left[E_{1}\right]|=\frac{q_{h}^{2}}{2.|Hash|} \end{array}$$ (3)

**Game** *G*_3_: In this game, *G*_3_ simulates the *CorruptSC* oracle which models the smart card lost attack. Since the chosen password has low entropy, A may try online dictionary attack with the information obtained from the smart card. In addition, A may try to obtain biometrics key *B*_*i*_ from information collected from the smart card *SC*_*i*_. Our protocol P uses BioHash, which extracts at most *l* nearly random bits, therefore the probability of guessing biometric key *B*_*i*_ ∈ {0, 1}^*l*^ by A is approximated as $\frac{1}{2^{l}}$. If the number of wrong password inputs is limited by the system, probabilities can be estimated as follows:

$$\begin{array}{c} |pr\left[E_{3}\right]-pr\left[E_{2}\right]|\leq \frac{q_{send}}{2^{l}.\left|D\right|} \end{array}$$ (4)

**Game** *G*_4_: This game models an attack wherein A has to compute the real session key *SK*_*ij*_ = *h*(*n*_1_||*n*_2_||*K*||*ID*_*i*_) using *K*, *ID*_*i*_ from the eavesdropping messages {*M*_1_, *Z*_*i*_, *T*_1_} and {*M*_2_, *M*_3_, *T*_2_}. A can not compute *K* = *h*((*W*_*i*_ ⊕ *IDB*_*i*_) ⊕ *h*(*ID*_*i*_||*n*_1_)) and $(ID_{i}\left|\right|n_{1})=E_{Pri_{j}}\left(M_{1}\right)$ as *ID*_*i*_, *Pri*_*j*_ and *IDB*_*i*_ are unknown. A also needs to derive *n*_1_ and *n*_2_ from *M*_1_ and *M*_2_, respectively. We then have

$$\begin{array}{c} |pr\left[E_{4}\right]-pr\left[E_{3}\right]|\leq Adv^{RSA}\left(t\right) \end{array}$$ (5)

Additionally, since all session keys are random and independent and no information about the value of *c* is revealed to A, Then,

$$\begin{array}{c} pr\left[E_{4}\right]=\frac{1}{2} \end{array}$$ (6)

From Eqs (1)–(6), the following result is obtained:

$$\begin{array}{c} Adv_{p}^{ake}\leq \frac{q_{h}^{2}}{\left|Hash\right|}+\frac{q_{send}}{2^{l-1}.\left|D\right|}+2Adv^{RSA}\left(t\right) \end{array}$$ (7)

### 6.3 Informal security analysis

This subsection describes the security analysis of our scheme. To evaluate the security of the improved scheme, we assume that the adversary might access the smart card of legal user and extract the information stored in the smart card and intercept information transmitted over the public channel.

#### 6.3.1 Mutual authentication

After receiving the login request information from *U*_*i*_, *S*_*j*_ checks if *Z*_*i*_? = *h*(*n*_1_||*ID*_*i*_ ||*K*||*T*_1_) holds or not. The adversary who masquerades as the legal user cannot forge *Z*_*i*_ without knowing *ID*_*i*_ and the biometrics *BIO*_*i*_ of *U*_*i*_. Likewise, upon receiving the message *M*_3_, *U*_*i*_ checks *M*_3_? = *h*(*ID*_*i*_||*n*_1_||*n*_2_||*K*||*T*_2_), where *K* = *h*(*h*(*h*(*ID*_*i*_)|| *PSK*) ⊕ *h*(*ID*_*i*_||*n*_1_)), which requires the computation of *U*_*i*_’s identity *ID*_*i*_, the random number *n*_1_ and *PSK*. Only the server who has the private key *Pri*_*j*_ can compute *ID*_*i*_ and *n*_1_ so as to get the value of *K*. Hence only legal user can share the session key with corresponding server. Therefore, our proposed scheme can provide proper mutual authentication.

#### 6.3.2 Anonymity

In the proposed scheme, the login request message < *M*_1_, *Z*_*i*_, *T*_1_ > is dynamic for every login and does not disclose any information about *U*_*i*_, since it is associated with random number *n*_1_. The identity is protected by the encrypted message $M_{1}=E_{{pub}_{j}}(ID_{i}\left|\right|n_{1})$ using *Pub*_*j*_. The adversary cannot obtain *ID*_*i*_ without having the knowledge of *Pri*_*j*_. In addition, the unauthorized server cannot decrypt the user’s authentication message successfully since it does not own the private key *Pri*_*j*_. As a result, the user’s real identity cannot be retrieved. Thus our protocol can achieve the anonymity property of users as well as protect the privacy of users.

#### 6.3.3 Off-line password guessing attack

An adversary may try to guess the password *PW*_*i*_ from the extracted smart card stored parameters < *V*_*i*_, *W*_*i*_, *h*(⋅), *H*(⋅) >. The stored parameter contains the password *PW*_*i*_ in the form *V*_*i*_ = *h*(*h*(*ID*_*i*_)||*PWD*_*i*_) where *PWD*_*i*_ = *h*(*PW*_*i*_||*H*(*BIO*_*i*_)). An adversary attempts to verify the condition *V*_*i*_? = *h*(*h*(*ID*_*i*_)||*h*(*PW*_*i*_||*H*(*BIO*_*i*_)) while constantly guessing *PW*_*i*_. Adversary needs the value of *ID*_*i*_ and *BIO*_*i*_ of *U*_*i*_ in order to achieve the password guessing attack. However, the value of *BIO*_*i*_ is nowhere stored and an adversary cannot get the value of *ID*_*i*_ without knowing the private key *Pri*_*j*_. As a result, the adversary cannot guess the correct password *PW*_*i*_. Therefore, our proposed improved protocol can withstand this kind of attack.

#### 6.3.4 Insider attack

In our proposed protocol, *U*_*i*_ does not send his *ID*_*i*_, password *PW*_*i*_ or his biometrics *BIO*_*i*_ in plain text during user registration phase. *U*_*i*_ submits only *h*(*ID*_*i*_), *IDB*_*i*_ and *PWD*_*i*_ to *RS* instead of original credentials, where *PWD*_*i*_ = *h*(*PW*_*i*_||*H*(*BIO*_*i*_)), *IDB*_*i*_ = *h*(*ID*_*i*_||*H*(*BIO*_*i*_)). Hence, an insider cannot obtain the original sensitive information of any user. On the other hand, the authentication of entities is being done by verifying message like *Z*_*i*_? = *h*(*n*_1_||*ID*_*i*_||*K*||*T*_1_) in which *ID*_*i*_ is necessary. Moreover, *RS* doesn’t participate in the authentication process. Therefore, the proposed protocol attains resistance to insider attack.

#### 6.3.5 Stolen smart card attack

The adversary can extract the information < *V*_*i*_, *W*_*i*_, *h*(⋅), *H*(⋅) > stored in the smart card by means of power analysis. Assume a legal user’s smart card is stolen by an adversary and the stored information < *V*_*i*_, *W*_*i*_, *h*(⋅), *H*(⋅) > on it are extracted. Then, the adversary may try to get *ID*_*i*_, *PW*_*i*_, *BIO*_*i*_ from the extracted information. However, adversary cannot obtain any valuable information from these values, where *V*_*i*_ = *h*(*h*(*ID*_*i*_)||*PWD*_*i*_) and *W*_*i*_ = *h*(*h*(*ID*_*i*_)||*PSK*) ⊕ *IDB*_*i*_, since all the important parameters such as *ID*_*i*_ and *PW*_*i*_ are protected by a one-way hash function. The adversary cannot obtain any login information using the smart card stored parameters *V*_*i*_ and *W*_*i*_. At the same time guessing the real identity *ID*_*i*_ and password *PW*_*i*_ is impractical. Therefore, the proposed protocol is secure against smart card stolen attack.

#### 6.3.6 Replay attack

If an adversary has intercepted all the communication message < *M*_1_, *Z*_*i*_, *T*_1_ > and < *M*_2_, *M*_3_, *T*_2_ >, he tries to replay them to *U*_*i*_ or *S*_*j*_ to masquerade as a legal user. However, once the message is replayed, the server can immediately detect the attack and reject the request due to the apply of timestamp. Hence, our scheme is secure against replay attack.

### 6.4 No verification table

In the proposed scheme, the registration server and application servers do not store the password and the biometrics database of the user. Therefore, even if an adversary steals the information stored in *RS*, he still cannot get *ID*_*i*_, *PW*_*i*_, *BIO*_*i*_ or other valid information of users. *S*_*j*_ does not store the password or the biometrics table of users as well. Therefore, even if an adversary steals the database from *RS*, he still cannot obtain user’s sensitive information of users.

#### 6.4.1 User masquerade attack

Assume an adversary steals a smart card from a legal user and wants to get service by perpetrating user impersonation attack. If an adversary forges messages so as to impersonate as *U*_*i*_, he needs to build a login request message < *M*_1_, *Z*_*i*_, *T*_1_ > firstly, where $M_{1}=E_{{Pub}_{j}}(ID_{i}\left|\right|n_{1})$, *Z*_*i*_ = *h*(*n*_1_||*ID*_*i*_||*K*||*T*_1_). Conversely, the adversary cannot compute the messages *M*_1_ and *Z*_*i*_ without user’s private information *ID*_*i*_ and *H*(*BIO*_*i*_). At the same time, the adversary has to go through login phase before sending login request information. During login phase, *SC* computes *PWD*_*i*_ = *h*(*PW*_*i*_||*H*(*BIO*_*i*_)) and then verifies if *V*_*i*_? = *h*(*ID*_*i*_||*PWD*_*i*_) is correct. Unless the adversary enters the correct credentials, the process will be terminated. Therefore, the adversary certainly requires *ID*_*i*_, *PW*_*i*_ and *BIO*_*i*_ for any furthermore computations. However, the probability of obtaining correct *ID*_*i*_, *PW*_*i*_ and *BIO*_*i*_ is negligible.

#### 6.4.2 Server impersonation attack

Unlike Moon *et al.*’s protocol, the server *S*_*j*_ not only keeps unique long-term key *PSK*, but also contains the key pair < *Pub*_*j*_, *Pri*_*j*_ >. Note that the key pair of each server is distinctive, and *Pri*_*j*_ is known to only server *S*_*j*_. Consider a scenario where an adversary captures < *M*_1_, *Z*_*i*_, *T*_1_ > and tries to impersonate valid server by responding with message < *M*_2_, *M*_3_, *T*_2_ >. The values of *ID*_*i*_, *K* and *n*_1_ are prerequisite. However, adversary cannot yield either of the values without having the knowledge of *Pri*_*j*_. Though, the adversary cannot get the right values of *ID*_*i*_, *K* and *n*_1_, if the adversary forges the massage < *M*_2_, *M*_3_, *T*_2_ >. Upon receiving the response message < *M*_2_, *M*_3_, *T*_2_ >, *U*_*i*_ can identify it as a malicious attempt due to the non-equivalence of message $M_{3}^{\prime }?=M_{3}$. Thus, our proposed protocol is secure against server impersonation attack.

#### 6.4.3 Forward secrecy

In our improved protocol, the session key is *SK*_*ij*_ = *h*(*n*_1_||*n*_2_||*K*||*ID*_*i*_), and the values of the long term private key of the servers vary from server to server and are not shared with any registered *U*_*i*_. Assume that the adversary has obtained the long term key *PSK*, he still cannot compute a valid session key without the secret parameters *ID*_*i*_ and *n*_1_, which are protected by *Pub*_*j*_ and are decryptable only with *Pri*_*j*_. Moreover, the parameters *n*_1_ and *n*_2_ are random for each session. Therefore, the session key is considered to be safe even though the long term private key of the server is compromised.

## 7 Functional and performance analysis

In this section, we compare our proposed scheme with the other related schemes in term of the functionality, including Chuang *et al.*’s scheme, Mishra *et al.*’s scheme and Lu *et al.*’s scheme.

### 7.1 Functional analysis

We perform a comparative analysis of previous schemes, which is illustrated in Table 3. From the table, we can find that the proposed scheme is more secure and provides more functionality requirements than the other related schemes. Moreover, the proposed scheme achieves all resistance requirements.

**Table 3:** Functionality comparison.

Scheme	Chuang [27]	Mishra [28]	Lu [29]	Lu [31]	Moon [30]	our
Provide mutual authentication	No	Yes	Yes	Yes	Yes	Yes
User anonymity	Yes	Yes	No	No	No	Yes
Resist insider attack	Yes	Yes	Yes	Yes	No	Yes
Resist off-line guessing attack	Yes	Yes	Yes	Yes	No	Yes
Resist smart card theft attack	No	Yes	Yes	Yes	Yes	Yes
Resist replay attack	No	No	No	Yes	Yes	Yes
Resist Impersonation attack	No	No	No	No	No	Yes
Session key agreement	Yes	Yes	Yes	Yes	Yes	Yes
Provides Forward secrecy	Yes	No	Yes	Yes	Yes	Yes
Efficient password change phase	No	No	Yes	Yes	Yes	Yes
Resist verifier attack	Yes	Yes	Yes	Yes	Yes	Yes

### 7.2 Performance analysis

Now we compare the computational costs and execution time between the proposed scheme and the other related schemes. For the evaluation of the computational costs, let *T*_*h*_, *T*_*Re*_, *T*_*Rd*_, *T*_*sym*_ and *T*_*epm*_ refer to the execution time of one-way hash, *RSA* encryption, *RSA* decryption, symmetric key encryption/decryption operation and complexity of executing an elliptic curve point multiplication operation. According to Kilinc *et al.*’s [40] estimation, the average running time of *T*_*h*_ is about 0.0023ms, *T*_*Re*_ is 3.8500ms, *T*_*Rd*_ is 0.1925ms, *T*_*sym*_ is 0.1303 ms and *T*_*epm*_ is 2.229ms. Table 4 illustrates the comparative performance of our improved scheme and previously proposed schemes.

**Table 4:** Computation costs comparison.

Scheme	Login	Authentication	Total	Time(ms)
Chuang *et al.*’s [27]	4*T*_*h*_	13*T*_*h*_	17*T*_*h*_	0.0391
Mishra *et al.*’s [28]	4*T*_*h*_	11*T*_*h*_	15*T*_*h*_	0.0345
Lu *et al.*’s [29]	6*T*_*h*_	12*T*_*h*_	18*T*_*h*_	0.0414
Moon *et al.*’s [30]	5*T*_*h*_	13*T*_*h*_	18*T*_*h*_	0.0414
Lu *et al.*’s [31]	4*T*_*h*_ + 3*T*_*Re*_	14*T*_*h*_ + 3*T*_*Rd*_	18*T*_*h*_ + 3*T*_*Re*_ + 3*T*_*Rd*_	12.1689
Mishra’s [32]	6*T*_*h*_ + 2*T*_*epm*_	10*T*_*h*_ + 1*T*_*epm*_	16*T*_*h*_ + 3*T*_*epm*_	6.7148
Chaudhry’s [33]	2*T*_*h*_ + 3*T*_*epm*_	6*T*_*h*_ + 5*T*_*epm*_	8*T*_*h*_ + 8*T*_*epm*_	17.8504
Jiang’s [34]	3*T*_*h*_ + 1*T*_*epm*_ + *T*_*sym*_	6*T*_*h*_ + 3*T*_*epm*_ + 3*T*_*sym*_	9*T*_*h*_ + 6*T*_*epm*_ + 4*T*_*sym*_	13.9159
our scheme	7*T*_*h*_ + 1*T*_*Re*_	11*T*_*h*_ + 1*T*_*Rd*_	18*T*_*h*_ + *T*_*Re*_ + *T*_*Rd*_	4.0866

The time consumption of our proposed scheme and of the other related schemes is listed in Table 4. The results shows that the proposed scheme is the most computationally inexpensive one among those schemes based on public key cryptography [31–34]. Note that although our proposed scheme costs more time than rest of the schemes [27–30], it is more secure than these schemes. To sum up, only the proposed scheme provides both the computation efficiency to accomplish mutual authentication and key agreement, and the basic security properties against the known threats. The rest of schemes either are vulnerable to various attacks [27–31], or need more time than our scheme [31–34].

## 8 Conclusion

In this paper, we firstly analyzed the security of Moon *et al*’s scheme, and demonstrated that their scheme is vulnerable to the known internal attack, guess attack and impersonation attack. Moreover, their scheme is found not anonymous for the user. To withstand these drawbacks, we proposed an improved biometric-based authentication scheme for multi-server environment and proved that the improved scheme provides secure authentication through the formal security analysis using Burrows-Abadi-Needham logic (BAN-logic) and random oracle model. Moreover, we have shown that our scheme is robust against all known attacks through the informal security analysis. The functional and performance analysis shows that the improved scheme has the best secure functionality and is computational efficient.
